# Supplementary material for: Hot spot prediction in protein-protein interactions by an ensemble system
Source: BMC Syst Biol. 2018 Dec 31;12(Suppl 9):132. doi: 10.1186/s12918-018-0665-8 (PMC6311905; doi:10.1186/s12918-018-0665-8)
Supplement: Supplementary file 2 — All relevant MATLAB code. A simple Matlab implement of our predictor. (ZIP 19,742 kb) [file 12918_2018_665_MOESM2_ESM.zip › Additional file 2/Read ASA/ASA website/dbMPIKT/5E6PA.html]

 NetSurfP - Protein Surface Accessibility and Secondary Structure Predictions

  

|  |  |  |
| --- | --- | --- |
|  |  | NetSurfP - Protein Surface Accessibility and Secondary Structure Predictions  Technical University of Denmark |

  


---

```
# For publication of results, please cite:
# A generic method for assignment of reliability scores applied to solvent accessibility predictions.
# Bent Petersen, Thomas Nordahl Petersen, Pernille Andersen, Morten Nielsen and Claus Lundegaard
# BMC Structural Biology 2009, 9:51 doi:10.1186/1472-6807-9-51
#
# Column 1: Class assignment - B for buried or E for Exposed - Threshold: 25% exposure, but not based on RSA
# Column 2: Amino acid
# Column 3: Sequence name
# Column 4: Amino acid number
# Column 5: Relative Surface Accessibility - RSA
# Column 6: Absolute Surface Accessibility
# Column 7: Z-fit score for RSA prediction
# Column 8: Probability for Alpha-Helix
# Column 9: Probability for Beta-strand
# Column 10: Probability for Coil
E G  5E6PA                  1    0.826  64.983  -0.719   0.003   0.003   0.994
E P  5E6PA                  2    0.586  83.139  -1.142   0.058   0.017   0.925
E H  5E6PA                  3    0.404  73.415  -1.699   0.181   0.016   0.803
E M  5E6PA                  4    0.462  92.526   0.194   0.339   0.016   0.645
E R  5E6PA                  5    0.459 105.203  -0.284   0.430   0.016   0.555
E K  5E6PA                  6    0.438  90.014   0.136   0.354   0.048   0.598
E S  5E6PA                  7    0.376  44.091  -0.434   0.430   0.016   0.555
E Q  5E6PA                  8    0.564 100.713   0.412   0.879   0.010   0.111
E Q  5E6PA                  9    0.591 105.517   0.880   0.923   0.002   0.076
B A  5E6PA                 10    0.243  26.823  -0.574   0.970   0.001   0.030
E E  5E6PA                 11    0.435  75.925  -0.330   0.970   0.001   0.030
E R  5E6PA                 12    0.517 118.462   0.388   0.970   0.001   0.030
E E  5E6PA                 13    0.406  70.946   0.743   0.970   0.001   0.030
B Y  5E6PA                 14    0.123  26.264   0.793   0.988   0.000   0.012
E E  5E6PA                 15    0.521  90.966   1.086   0.988   0.000   0.012
E K  5E6PA                 16    0.494 101.636   1.465   0.988   0.000   0.012
B I  5E6PA                 17    0.074  13.764   0.361   0.988   0.000   0.012
E K  5E6PA                 18    0.349  71.728   1.074   0.988   0.000   0.012
E S  5E6PA                 19    0.488  57.147   0.967   0.988   0.000   0.012
B Q  5E6PA                 20    0.229  40.828   0.906   0.988   0.000   0.012
B L  5E6PA                 21    0.074  13.604   0.283   0.970   0.001   0.030
E E  5E6PA                 22    0.486  84.852   1.266   0.970   0.001   0.030
E G  5E6PA                 23    0.445  34.982   0.910   0.970   0.001   0.030
B L  5E6PA                 24    0.062  11.279   0.643   0.970   0.001   0.030
B E  5E6PA                 25    0.220  38.521  -0.077   0.970   0.001   0.030
E E  5E6PA                 26    0.502  87.717   0.319   0.970   0.001   0.030
E S  5E6PA                 27    0.407  47.677   0.943   0.970   0.001   0.030
B V  5E6PA                 28    0.038   5.856  -0.470   0.970   0.001   0.030
E R  5E6PA                 29    0.238  54.502   0.512   0.970   0.001   0.030
E D  5E6PA                 30    0.591  85.134   0.737   0.970   0.001   0.030
E R  5E6PA                 31    0.337  77.104   1.468   0.970   0.001   0.030
B C  5E6PA                 32    0.018   2.541   1.123   0.970   0.001   0.030
E K  5E6PA                 33    0.343  70.514   1.564   0.970   0.001   0.030
E K  5E6PA                 34    0.577 118.607   1.544   0.923   0.002   0.076
B E  5E6PA                 35    0.113  19.759   0.368   0.923   0.002   0.076
B F  5E6PA                 36    0.049   9.895   0.375   0.858   0.002   0.139
E T  5E6PA                 37    0.300  41.638   0.179   0.858   0.002   0.139
E D  5E6PA                 38    0.438  63.044   0.428   0.879   0.010   0.111
B L  5E6PA                 39    0.088  16.113   0.659   0.802   0.014   0.185
B M  5E6PA                 40    0.186  37.139  -0.175   0.802   0.014   0.185
E I  5E6PA                 41    0.327  60.587  -0.138   0.717   0.014   0.269
E E  5E6PA                 42    0.340  59.381  -0.388   0.717   0.014   0.269
B M  5E6PA                 43    0.135  27.074   0.385   0.717   0.014   0.269
E E  5E6PA                 44    0.471  82.301  -0.693   0.622   0.015   0.363
E D  5E6PA                 45    0.507  73.044  -0.207   0.522   0.016   0.462
B Q  5E6PA                 46    0.141  25.111   0.594   0.622   0.015   0.363
E T  5E6PA                 47    0.425  59.003  -0.168   0.622   0.015   0.363
E N  5E6PA                 48    0.729 106.726   0.125   0.430   0.016   0.555
E D  5E6PA                 49    0.437  62.914   0.320   0.339   0.016   0.645
B V  5E6PA                 50    0.117  17.998  -0.687   0.257   0.016   0.727
E H  5E6PA                 51    0.603 109.631  -0.271   0.115   0.016   0.868
E E  5E6PA                 52    0.620 108.262  -0.983   0.058   0.017   0.925
E A  5E6PA                 53    0.333  36.686  -0.885   0.018   0.019   0.964
E G  5E6PA                 54    0.350  27.576  -0.758   0.005   0.045   0.951
B I  5E6PA                 55    0.087  16.151   0.632   0.004   0.085   0.910
B P  5E6PA                 56    0.158  22.392  -0.164   0.021   0.279   0.699
B T  5E6PA                 57    0.173  23.995  -0.787   0.021   0.451   0.528
B L  5E6PA                 58    0.130  23.876  -0.030   0.021   0.451   0.528
E D  5E6PA                 59    0.398  57.280  -0.326   0.064   0.216   0.721
B Y  5E6PA                 60    0.198  42.291  -0.227   0.184   0.043   0.773
E K  5E6PA                 61    0.366  75.204   1.149   0.354   0.048   0.598
E T  5E6PA                 62    0.322  44.648   0.421   0.428   0.171   0.402
B Y  5E6PA                 63    0.099  21.092   0.761   0.453   0.248   0.299
B T  5E6PA                 64    0.039   5.354  -0.168   0.478   0.309   0.214
B D  5E6PA                 65    0.208  29.915   0.284   0.453   0.248   0.299
B R  5E6PA                 66    0.145  33.228   0.062   0.352   0.332   0.316
B V  5E6PA                 67    0.046   7.055   0.277   0.231   0.330   0.439
B F  5E6PA                 68    0.043   8.610  -0.376   0.135   0.317   0.548
B F  5E6PA                 69    0.054  10.778  -0.522   0.064   0.216   0.721
B L  5E6PA                 70    0.188  34.350  -0.346   0.056   0.142   0.802
E P  5E6PA                 71    0.399  56.604  -1.190   0.018   0.047   0.935
E S  5E6PA                 72    0.446  52.330  -1.169   0.018   0.047   0.935
E K  5E6PA                 73    0.506 104.125  -1.076   0.058   0.017   0.925
E D  5E6PA                 74    0.472  68.087  -1.201   0.058   0.017   0.925
B G  5E6PA                 75    0.271  21.343  -1.528   0.058   0.017   0.925
E D  5E6PA                 76    0.312  45.017  -1.335   0.113   0.043   0.844
E K  5E6PA                 77    0.582 119.738  -0.021   0.113   0.087   0.800
E D  5E6PA                 78    0.407  58.634  -0.739   0.056   0.142   0.802
B V  5E6PA                 79    0.169  25.960   0.769   0.056   0.142   0.802
B M  5E6PA                 80    0.207  41.361  -0.944   0.066   0.296   0.638
B I  5E6PA                 81    0.130  24.124   0.261   0.021   0.279   0.699
B T  5E6PA                 82    0.282  39.155  -0.069   0.020   0.205   0.775
E G  5E6PA                 83    0.508  39.956  -0.074   0.018   0.088   0.893
E K  5E6PA                 84    0.514 105.730  -0.291   0.018   0.047   0.935
B L  5E6PA                 85    0.241  44.072  -0.838   0.018   0.019   0.964
E D  5E6PA                 86    0.696 100.236  -0.860   0.018   0.019   0.964
E I  5E6PA                 87    0.342  63.233  -0.190   0.058   0.017   0.925
E P  5E6PA                 88    0.385  54.575  -1.530   0.109   0.005   0.886
E E  5E6PA                 89    0.637 111.336  -1.166   0.176   0.004   0.820
E S  5E6PA                 90    0.408  47.876  -1.258   0.321   0.003   0.675
B R  5E6PA                 91    0.275  62.906  -0.315   0.406   0.004   0.590
E R  5E6PA                 92    0.465 106.531   0.388   0.694   0.003   0.303
E P  5E6PA                 93    0.634  89.908   0.564   0.782   0.003   0.216
B I  5E6PA                 94    0.235  43.512   0.391   0.858   0.002   0.139
B V  5E6PA                 95    0.080  12.296   0.668   0.970   0.001   0.030
E E  5E6PA                 96    0.485  84.817   1.484   0.970   0.001   0.030
E Q  5E6PA                 97    0.416  74.387   1.883   0.970   0.001   0.030
B A  5E6PA                 98    0.028   3.130   0.132   0.970   0.001   0.030
B L  5E6PA                 99    0.036   6.665   0.238   0.970   0.001   0.030
E Y  5E6PA                100    0.331  70.799   0.729   0.970   0.001   0.030
B Q  5E6PA                101    0.189  33.684   0.878   0.970   0.001   0.030
B F  5E6PA                102    0.030   5.941   0.022   0.970   0.001   0.030
B S  5E6PA                103    0.162  18.963  -0.386   0.970   0.001   0.030
E N  5E6PA                104    0.416  60.829   0.673   0.970   0.001   0.030
B L  5E6PA                105    0.094  17.175   0.141   0.923   0.002   0.076
B L  5E6PA                106    0.046   8.459  -0.179   0.782   0.003   0.216
E N  5E6PA                107    0.380  55.676  -0.269   0.406   0.004   0.590
E S  5E6PA                108    0.427  50.091  -0.597   0.109   0.005   0.886
E K  5E6PA                109    0.368  75.595  -0.264   0.176   0.004   0.820
B S  5E6PA                110    0.264  30.917  -0.486   0.430   0.016   0.555
B F  5E6PA                111    0.087  17.501   0.403   0.701   0.107   0.192
B L  5E6PA                112    0.040   7.416   0.160   0.807   0.137   0.056
B I  5E6PA                113    0.078  14.430   0.423   0.807   0.137   0.056
B N  5E6PA                114    0.196  28.665   0.931   0.867   0.107   0.026
B F  5E6PA                115    0.066  13.206   0.519   0.746   0.197   0.057
B I  5E6PA                116    0.044   8.103   0.204   0.725   0.163   0.112
E H  5E6PA                117    0.296  53.824   0.389   0.701   0.107   0.192
B T  5E6PA                118    0.179  24.869  -0.470   0.751   0.050   0.199
B L  5E6PA                119    0.089  16.241   0.812   0.622   0.015   0.363
E E  5E6PA                120    0.406  70.928   0.442   0.622   0.015   0.363
E N  5E6PA                121    0.614  89.875  -0.069   0.522   0.016   0.462
E Q  5E6PA                122    0.376  67.225   0.006   0.257   0.016   0.727
E R  5E6PA                123    0.498 114.019  -0.084   0.184   0.043   0.773
E E  5E6PA                124    0.560  97.780  -0.201   0.113   0.043   0.844
B F  5E6PA                125    0.186  37.390  -0.417   0.053   0.043   0.903
E S  5E6PA                126    0.361  42.321  -0.247   0.113   0.043   0.844
E A  5E6PA                127    0.409  45.105  -0.938   0.354   0.048   0.598
E R  5E6PA                128    0.429  98.241  -0.114   0.561   0.047   0.393
B A  5E6PA                129    0.193  21.269  -1.934   0.622   0.015   0.363
B K  5E6PA                130    0.227  46.694   0.168   0.802   0.014   0.185
B V  5E6PA                131    0.075  11.558  -1.457   0.831   0.044   0.125
B Y  5E6PA                132    0.123  26.328   0.534   0.879   0.010   0.111
B F  5E6PA                133    0.032   6.362  -0.000   0.938   0.007   0.055
B A  5E6PA                134    0.042   4.606  -0.927   0.938   0.007   0.055
B S  5E6PA                135    0.079   9.294  -1.169   0.970   0.001   0.030
B L  5E6PA                136    0.041   7.599  -0.063   0.970   0.001   0.030
B L  5E6PA                137    0.057  10.437   0.474   0.970   0.001   0.030
B T  5E6PA                138    0.081  11.207   0.336   0.970   0.001   0.030
B V  5E6PA                139    0.043   6.594   0.546   0.970   0.001   0.030
B A  5E6PA                140    0.065   7.108  -0.183   0.938   0.007   0.055
B L  5E6PA                141    0.097  17.816  -0.438   0.879   0.010   0.111
E H  5E6PA                142    0.314  57.062  -0.083   0.600   0.003   0.397
E G  5E6PA                143    0.427  33.573  -1.483   0.246   0.004   0.750
E K  5E6PA                144    0.385  79.256  -0.470   0.246   0.004   0.750
B L  5E6PA                145    0.167  30.633  -0.345   0.502   0.002   0.495
E E  5E6PA                146    0.474  82.755  -0.187   0.717   0.014   0.269
B Y  5E6PA                147    0.213  45.454   0.480   0.802   0.014   0.185
B Y  5E6PA                148    0.048  10.322   0.172   0.923   0.002   0.076
B T  5E6PA                149    0.146  20.181  -0.229   0.970   0.001   0.030
E D  5E6PA                150    0.369  53.245  -0.128   0.970   0.001   0.030
B I  5E6PA                151    0.060  11.156   0.692   0.970   0.001   0.030
B M  5E6PA                152    0.033   6.563   0.408   0.988   0.000   0.012
B R  5E6PA                153    0.226  51.708   1.163   0.988   0.000   0.012
E T  5E6PA                154    0.302  41.915   1.286   0.970   0.001   0.030
B L  5E6PA                155    0.070  12.854   0.849   0.970   0.001   0.030
B F  5E6PA                156    0.041   8.309  -0.272   0.970   0.001   0.030
E L  5E6PA                157    0.256  46.874   0.855   0.970   0.001   0.030
E E  5E6PA                158    0.438  76.484   0.925   0.970   0.001   0.030
B L  5E6PA                159    0.099  18.054   0.903   0.970   0.001   0.030
B M  5E6PA                160    0.049   9.845   0.561   0.970   0.001   0.030
E E  5E6PA                161    0.452  78.982   1.361   0.970   0.001   0.030
E Q  5E6PA                162    0.557  99.534   1.117   0.923   0.002   0.076
B Y  5E6PA                163    0.164  35.068  -0.248   0.858   0.002   0.139
B V  5E6PA                164    0.159  24.469  -0.478   0.802   0.014   0.185
E V  5E6PA                165    0.584  89.761   0.295   0.622   0.015   0.363
E A  5E6PA                166    0.533  58.748  -0.544   0.430   0.016   0.555
E K  5E6PA                167    0.633 130.270  -0.267   0.257   0.016   0.727
E N  5E6PA                168    0.462  67.578  -0.419   0.058   0.017   0.925
B P  5E6PA                169    0.196  27.798  -0.487   0.052   0.084   0.864
E K  5E6PA                170    0.357  73.538   0.601   0.069   0.386   0.545
B L  5E6PA                171    0.120  21.935  -0.246   0.079   0.592   0.329
B M  5E6PA                172    0.126  25.273  -0.026   0.074   0.484   0.442
B L  5E6PA                173    0.047   8.624  -0.871   0.069   0.386   0.545
B R  5E6PA                174    0.240  54.983   0.175   0.125   0.227   0.648
B R  5E6PA                175    0.280  64.143  -0.567   0.113   0.043   0.844
B S  5E6PA                176    0.196  22.936  -0.844   0.257   0.016   0.727
E E  5E6PA                177    0.334  58.315  -0.038   0.879   0.010   0.111
E T  5E6PA                178    0.296  41.083   0.592   0.879   0.010   0.111
B V  5E6PA                179    0.060   9.222   0.955   0.938   0.007   0.055
B V  5E6PA                180    0.058   8.838   0.302   0.975   0.003   0.022
B E  5E6PA                181    0.243  42.417   0.137   0.970   0.001   0.030
E R  5E6PA                182    0.274  62.769   1.007   0.988   0.000   0.012
B M  5E6PA                183    0.057  11.446   0.730   0.970   0.001   0.030
B L  5E6PA                184    0.069  12.652  -0.085   0.970   0.001   0.030
B S  5E6PA                185    0.198  23.194  -0.586   0.970   0.001   0.030
B N  5E6PA                186    0.131  19.164  -0.238   0.923   0.002   0.076
B W  5E6PA                187    0.079  18.927  -0.017   0.970   0.001   0.030
B M  5E6PA                188    0.087  17.489  -0.093   0.970   0.001   0.030
B S  5E6PA                189    0.064   7.548  -0.430   0.970   0.001   0.030
B I  5E6PA                190    0.048   8.972   0.490   0.970   0.001   0.030
B C  5E6PA                191    0.051   7.118  -0.006   0.970   0.001   0.030
B L  5E6PA                192    0.041   7.489  -0.192   0.970   0.001   0.030
B Y  5E6PA                193    0.094  20.024   0.434   0.923   0.002   0.076
B Q  5E6PA                194    0.233  41.596   0.315   0.923   0.002   0.076
B Y  5E6PA                195    0.132  28.230   0.279   0.858   0.002   0.139
B L  5E6PA                196    0.041   7.452  -0.161   0.782   0.003   0.216
E K  5E6PA                197    0.289  59.427   1.119   0.782   0.003   0.216
E D  5E6PA                198    0.582  83.809   0.103   0.782   0.003   0.216
B S  5E6PA                199    0.174  20.393   0.376   0.502   0.002   0.495
B A  5E6PA                200    0.094  10.304  -0.712   0.406   0.004   0.590
E G  5E6PA                201    0.308  24.279  -0.798   0.246   0.004   0.750
E E  5E6PA                202    0.349  60.970   0.101   0.406   0.004   0.590
B P  5E6PA                203    0.207  29.402  -0.155   0.782   0.003   0.216
B L  5E6PA                204    0.049   9.009   0.544   0.923   0.002   0.076
B Y  5E6PA                205    0.127  27.204   0.006   0.970   0.001   0.030
B K  5E6PA                206    0.171  35.236   0.376   0.970   0.001   0.030
B L  5E6PA                207    0.028   5.090   0.571   0.988   0.000   0.012
B F  5E6PA                208    0.055  11.119   0.250   0.988   0.000   0.012
B K  5E6PA                209    0.207  42.662   0.751   0.988   0.000   0.012
B A  5E6PA                210    0.059   6.447  -0.702   0.988   0.000   0.012
B I  5E6PA                211    0.038   7.067  -0.278   0.970   0.001   0.030
E K  5E6PA                212    0.284  58.337   1.323   0.923   0.002   0.076
E H  5E6PA                213    0.361  65.593   0.816   0.858   0.002   0.139
B Q  5E6PA                214    0.143  25.468   0.067   0.802   0.014   0.185
B V  5E6PA                215    0.103  15.800  -0.119   0.622   0.015   0.363
E E  5E6PA                216    0.633 110.550  -0.194   0.430   0.016   0.555
E K  5E6PA                217    0.508 104.516   0.523   0.181   0.016   0.803
B G  5E6PA                218    0.266  20.918  -0.613   0.058   0.017   0.925
E P  5E6PA                219    0.322  45.720  -0.615   0.113   0.043   0.844
B V  5E6PA                220    0.190  29.188   0.195   0.278   0.093   0.628
E D  5E6PA                221    0.316  45.564  -0.508   0.307   0.165   0.527
B A  5E6PA                222    0.229  25.291  -0.185   0.352   0.332   0.316
B V  5E6PA                223    0.206  31.724  -0.113   0.252   0.423   0.325
E Q  5E6PA                224    0.350  62.474   0.043   0.231   0.330   0.439
E K  5E6PA                225    0.444  91.310  -0.913   0.216   0.235   0.548
E K  5E6PA                226    0.553 113.711   0.803   0.135   0.317   0.548
B A  5E6PA                227    0.098  10.767  -0.708   0.159   0.516   0.325
E K  5E6PA                228    0.330  67.860   0.814   0.169   0.612   0.219
B Y  5E6PA                229    0.145  30.986   0.668   0.159   0.516   0.325
B T  5E6PA                230    0.175  24.272  -0.105   0.159   0.516   0.325
B L  5E6PA                231    0.103  18.859   0.486   0.135   0.317   0.548
E N  5E6PA                232    0.337  49.278   0.184   0.118   0.150   0.732
E D  5E6PA                233    0.380  54.801  -0.722   0.191   0.086   0.723
E T  5E6PA                234    0.538  74.662  -0.455   0.184   0.043   0.773
E G  5E6PA                235    0.306  24.106   0.819   0.278   0.093   0.628
B L  5E6PA                236    0.105  19.171  -0.034   0.307   0.165   0.527
B L  5E6PA                237    0.105  19.171   0.527   0.199   0.152   0.649
E G  5E6PA                238    0.392  30.819   0.533   0.118   0.150   0.732
E D  5E6PA                239    0.497  71.546   0.116   0.125   0.227   0.648
E D  5E6PA                240    0.543  78.232   0.133   0.020   0.205   0.775
B V  5E6PA                241    0.141  21.687   1.095   0.021   0.451   0.528
E E  5E6PA                242    0.583 101.815   1.054   0.004   0.616   0.381
B Y  5E6PA                243    0.191  40.817   0.793   0.003   0.718   0.279
E A  5E6PA                244    0.473  52.180   1.225   0.002   0.816   0.182
E P  5E6PA                245    0.472  67.019   0.378   0.002   0.816   0.182
B L  5E6PA                246    0.106  19.354   0.433   0.001   0.900   0.099
B T  5E6PA                247    0.209  28.961   0.543   0.001   0.959   0.040
B V  5E6PA                248    0.051   7.839  -0.118   0.001   0.959   0.040
B S  5E6PA                249    0.266  31.187  -0.172   0.001   0.959   0.040
B V  5E6PA                250    0.031   4.842  -0.018   0.001   0.959   0.040
B I  5E6PA                251    0.113  20.905  -0.171   0.001   0.900   0.099
B V  5E6PA                252    0.187  28.788  -0.409   0.003   0.718   0.279
E Q  5E6PA                253    0.438  78.209  -0.464   0.004   0.197   0.799
E D  5E6PA                254    0.494  71.243  -1.833   0.005   0.015   0.979
E E  5E6PA                255    0.725 126.658  -0.795   0.005   0.015   0.979
E G  5E6PA                256    0.657  51.667  -2.224   0.005   0.015   0.979
E I  5E6PA                257    0.429  79.384  -1.110   0.005   0.045   0.951
E D  5E6PA                258    0.511  73.635  -0.740   0.005   0.262   0.733
E A  5E6PA                259    0.504  55.497   0.731   0.004   0.616   0.381
B I  5E6PA                260    0.096  17.797   0.436   0.002   0.816   0.182
E P  5E6PA                261    0.314  44.628   0.324   0.002   0.816   0.182
B V  5E6PA                262    0.042   6.425   0.471   0.001   0.900   0.099
E K  5E6PA                263    0.351  72.242   1.495   0.001   0.900   0.099
B V  5E6PA                264    0.052   8.054  -0.357   0.018   0.846   0.136
B L  5E6PA                265    0.140  25.726   0.294   0.023   0.655   0.322
B N  5E6PA                266    0.293  42.837  -0.848   0.021   0.451   0.528
B C  5E6PA                267    0.115  16.160   0.124   0.056   0.142   0.802
B D  5E6PA                268    0.330  47.611  -1.142   0.184   0.043   0.773
B T  5E6PA                269    0.179  24.855  -0.266   0.268   0.043   0.689
B I  5E6PA                270    0.113  20.886   0.449   0.622   0.015   0.363
E S  5E6PA                271    0.335  39.239   0.128   0.717   0.014   0.269
E Q  5E6PA                272    0.321  57.313   0.727   0.879   0.010   0.111
B V  5E6PA                273    0.064   9.883   0.230   0.938   0.007   0.055
B K  5E6PA                274    0.172  35.463   0.627   0.970   0.001   0.030
E E  5E6PA                275    0.429  74.894   0.782   0.970   0.001   0.030
E K  5E6PA                276    0.285  58.707   1.571   0.970   0.001   0.030
B I  5E6PA                277    0.039   7.252   0.261   0.970   0.001   0.030
B I  5E6PA                278    0.090  16.576   0.835   0.970   0.001   0.030
E D  5E6PA                279    0.424  61.026   0.754   0.970   0.001   0.030
B Q  5E6PA                280    0.181  32.255   0.379   0.970   0.001   0.030
B V  5E6PA                281    0.086  13.203   0.275   0.923   0.002   0.076
B Y  5E6PA                282    0.187  39.898   0.224   0.802   0.014   0.185
E R  5E6PA                283    0.525 120.248   0.074   0.522   0.016   0.462
E T  5E6PA                284    0.706  97.881  -0.680   0.181   0.016   0.803
B Q  5E6PA                285    0.235  41.989   0.220   0.018   0.019   0.964
E P  5E6PA                286    0.446  63.259  -1.184   0.018   0.047   0.935
B C  5E6PA                287    0.190  26.732  -0.296   0.019   0.141   0.840
E S  5E6PA                288    0.298  34.879  -1.696   0.020   0.205   0.775
E C  5E6PA                289    0.300  42.120  -1.324   0.019   0.141   0.840
E W  5E6PA                290    0.319  76.623  -0.201   0.018   0.047   0.935
B P  5E6PA                291    0.327  46.373  -1.105   0.018   0.047   0.935
E K  5E6PA                292    0.527 108.342   0.783   0.018   0.047   0.935
B P  5E6PA                293    0.163  23.116  -1.092   0.018   0.047   0.935
E D  5E6PA                294    0.522  75.177  -0.441   0.019   0.141   0.840
E S  5E6PA                295    0.378  44.255  -0.195   0.004   0.420   0.576
B V  5E6PA                296    0.068  10.452   0.306   0.003   0.718   0.279
E V  5E6PA                297    0.280  43.082   0.997   0.003   0.718   0.279
B L  5E6PA                298    0.068  12.432   1.044   0.001   0.900   0.099
E E  5E6PA                299    0.270  47.099   0.443   0.001   0.900   0.099
B W  5E6PA                300    0.164  39.538   0.467   0.001   0.900   0.099
E R  5E6PA                301    0.279  63.937   0.944   0.003   0.718   0.279
E P  5E6PA                302    0.453  64.309  -0.605   0.004   0.197   0.799
E G  5E6PA                303    0.588  46.315  -1.970   0.005   0.015   0.979
E S  5E6PA                304    0.418  48.978  -1.132   0.018   0.047   0.935
E T  5E6PA                305    0.557  77.284  -0.181   0.020   0.205   0.775
B A  5E6PA                306    0.279  30.735  -1.161   0.021   0.451   0.528
E Q  5E6PA                307    0.360  64.350   0.470   0.022   0.552   0.426
B I  5E6PA                308    0.266  49.302  -0.227   0.023   0.655   0.322
B L  5E6PA                309    0.155  28.344  -0.051   0.022   0.552   0.426
E S  5E6PA                310    0.404  47.337   0.155   0.022   0.359   0.619
E D  5E6PA                311    0.454  65.465  -0.799   0.021   0.279   0.699
E L  5E6PA                312    0.397  72.709  -0.668   0.019   0.141   0.840
E D  5E6PA                313    0.441  63.476  -0.800   0.018   0.088   0.893
E L  5E6PA                314    0.532  97.336  -0.347   0.052   0.084   0.864
E T  5E6PA                315    0.319  44.273  -0.993   0.052   0.084   0.864
E S  5E6PA                316    0.342  40.071  -0.589   0.064   0.216   0.721
E Q  5E6PA                317    0.496  88.586   0.674   0.069   0.386   0.545
E R  5E6PA                318    0.466 106.691   0.367   0.074   0.484   0.442
E E  5E6PA                319    0.679 118.691  -0.381   0.022   0.359   0.619
E G  5E6PA                320    0.510  40.137  -2.428   0.018   0.088   0.893
E R  5E6PA                321    0.611 139.965   0.112   0.018   0.047   0.935
B W  5E6PA                322    0.267  64.141  -0.545   0.021   0.279   0.699
E K  5E6PA                323    0.364  74.772   0.754   0.022   0.552   0.426
E R  5E6PA                324    0.345  79.074   0.486   0.022   0.552   0.426
B I  5E6PA                325    0.079  14.670   0.187   0.021   0.451   0.528
B N  5E6PA                326    0.230  33.731  -0.509   0.022   0.359   0.619
B T  5E6PA                327    0.181  25.049  -0.507   0.020   0.205   0.775
B L  5E6PA                328    0.208  38.030  -1.230   0.020   0.205   0.775
E M  5E6PA                329    0.372  74.357  -0.669   0.020   0.205   0.775
E H  5E6PA                330    0.320  58.208  -0.870   0.022   0.359   0.619
B Y  5E6PA                331    0.168  35.880   0.190   0.022   0.359   0.619
E N  5E6PA                332    0.509  74.503   0.245   0.021   0.279   0.699
B V  5E6PA                333    0.051   7.869   0.726   0.020   0.205   0.775
E R  5E6PA                334    0.535 122.629   0.462   0.018   0.088   0.893
E D  5E6PA                335    0.509  73.332  -0.780   0.018   0.047   0.935
E G  5E6PA                336    0.347  27.348  -1.241   0.018   0.088   0.893
B A  5E6PA                337    0.098  10.800  -0.364   0.005   0.336   0.660
B T  5E6PA                338    0.175  24.342   0.203   0.002   0.816   0.182
B L  5E6PA                339    0.057  10.363   0.658   0.001   0.900   0.099
B I  5E6PA                340    0.061  11.248  -1.174   0.001   0.900   0.099
B L  5E6PA                341    0.054   9.924   0.639   0.001   0.900   0.099
B S  5E6PA                342    0.066   7.782   0.090   0.002   0.816   0.182
B K  5E6PA                343    0.218  44.925   0.460   0.004   0.616   0.381
E V  5E6PA                344    0.328  50.460  -0.254   0.021   0.279   0.699
E G  5E6PA                345    0.421  33.133  -1.605   0.019   0.141   0.840
E V  5E6PA                346    0.376  57.760  -1.136   0.052   0.084   0.864
E S  5E6PA                347    0.425  49.787  -1.980   0.053   0.043   0.903
E Q  5E6PA                348    0.485  86.621  -1.617   0.053   0.043   0.903
E Q  5E6PA                349    0.489  87.318  -1.182   0.053   0.043   0.903
B P  5E6PA                350    0.237  33.687  -0.681   0.053   0.043   0.903
E E  5E6PA                351    0.432  75.505  -1.584   0.053   0.043   0.903
E D  5E6PA                352    0.516  74.384  -1.447   0.053   0.043   0.903
E S  5E6PA                353    0.399  46.810  -1.701   0.053   0.043   0.903
E Q  5E6PA                354    0.483  86.282  -1.665   0.053   0.043   0.903
E Q  5E6PA                355    0.574 102.463  -1.992   0.018   0.047   0.935
E D  5E6PA                356    0.415  59.816  -1.684   0.018   0.047   0.935
B L  5E6PA                357    0.199  36.437  -0.673   0.018   0.019   0.964
E P  5E6PA                358    0.381  54.036  -0.893   0.058   0.017   0.925
E G  5E6PA                359    0.499  39.311  -1.500   0.053   0.043   0.903
E E  5E6PA                360    0.575 100.452  -1.496   0.052   0.084   0.864
E R  5E6PA                361    0.442 101.218  -1.443   0.056   0.142   0.802
E H  5E6PA                362    0.345  62.701  -1.329   0.019   0.141   0.840
B A  5E6PA                363    0.279  30.779  -1.878   0.019   0.141   0.840
E L  5E6PA                364    0.328  60.112  -1.996   0.019   0.141   0.840
E L  5E6PA                365    0.364  66.685  -1.577   0.019   0.141   0.840
E E  5E6PA                366    0.389  67.958  -1.430   0.056   0.142   0.802
E E  5E6PA                367    0.607 106.095  -2.264   0.018   0.088   0.893
E E  5E6PA                368    0.529  92.364  -1.957   0.018   0.047   0.935
B N  5E6PA                369    0.272  39.777  -0.573   0.019   0.141   0.840
E R  5E6PA                370    0.287  65.700   0.460   0.022   0.552   0.426
B V  5E6PA                371    0.064   9.791   0.029   0.021   0.756   0.223
B W  5E6PA                372    0.066  15.825   0.600   0.018   0.846   0.136
B H  5E6PA                373    0.050   9.022  -0.825   0.018   0.846   0.136
B L  5E6PA                374    0.036   6.610  -0.405   0.021   0.756   0.223
B V  5E6PA                375    0.030   4.596  -0.617   0.004   0.616   0.381
E R  5E6PA                376    0.240  54.937   1.206   0.005   0.262   0.733
B P  5E6PA                377    0.135  19.199   0.604   0.005   0.045   0.951
E T  5E6PA                378    0.549  76.105  -0.082   0.018   0.019   0.964
E D  5E6PA                379    0.646  93.074  -1.228   0.058   0.017   0.925
E E  5E6PA                380    0.397  69.373  -1.139   0.115   0.016   0.868
E V  5E6PA                381    0.494  75.897  -1.362   0.257   0.016   0.727
E D  5E6PA                382    0.505  72.713  -1.272   0.257   0.016   0.727
E E  5E6PA                383    0.562  98.112  -0.671   0.257   0.016   0.727
E G  5E6PA                384    0.427  33.589  -1.542   0.184   0.043   0.773
E K  5E6PA                385    0.585 120.293  -0.342   0.184   0.043   0.773
E S  5E6PA                386    0.459  53.771  -1.145   0.113   0.087   0.800
E K  5E6PA                387    0.602 123.790  -1.379   0.052   0.084   0.864
E R  5E6PA                388    0.475 108.821  -1.654   0.053   0.043   0.903
E G  5E6PA                389    0.337  26.522  -2.739   0.018   0.047   0.935
E S  5E6PA                390    0.446  52.330  -1.801   0.018   0.047   0.935
E M  5E6PA                391    0.376  75.218  -2.082   0.018   0.019   0.964
E K  5E6PA                392    0.591 121.486  -0.910   0.058   0.017   0.925
E E  5E6PA                393    0.571  99.666  -1.845   0.257   0.016   0.727
E K  5E6PA                394    0.605 124.490  -0.456   0.430   0.016   0.555
E E  5E6PA                395    0.529  92.486  -0.879   0.522   0.016   0.462
E R  5E6PA                396    0.381  87.226   0.123   0.522   0.016   0.462
E T  5E6PA                397    0.497  68.934  -0.422   0.430   0.016   0.555
E K  5E6PA                398    0.464  95.507   0.291   0.455   0.046   0.498
B A  5E6PA                399    0.228  25.082  -0.455   0.354   0.048   0.598
B I  5E6PA                400    0.166  30.673   0.300   0.268   0.043   0.689
E T  5E6PA                401    0.362  50.168   0.015   0.354   0.048   0.598
E E  5E6PA                402    0.314  54.856  -0.661   0.502   0.102   0.396
B I  5E6PA                403    0.158  29.156   0.258   0.502   0.102   0.396
B Y  5E6PA                404    0.143  30.474  -0.004   0.502   0.102   0.396
B L  5E6PA                405    0.083  15.197   0.121   0.561   0.047   0.393
E T  5E6PA                406    0.290  40.181   0.104   0.561   0.047   0.393
B R  5E6PA                407    0.268  61.464   0.113   0.561   0.047   0.393
B L  5E6PA                408    0.124  22.759   0.050   0.561   0.047   0.393
B L  5E6PA                409    0.108  19.702   0.348   0.455   0.046   0.498
B S  5E6PA                410    0.244  28.609  -0.731   0.455   0.046   0.498
B V  5E6PA                411    0.200  30.786  -0.479   0.455   0.046   0.498
E K  5E6PA                412    0.312  64.076  -0.070   0.522   0.016   0.462
E G  5E6PA                413    0.336  26.412  -1.069   0.522   0.016   0.462
B T  5E6PA                414    0.207  28.669   0.187   0.622   0.015   0.363
B L  5E6PA                415    0.054   9.924  -0.068   0.802   0.014   0.185
E Q  5E6PA                416    0.300  53.526   0.683   0.802   0.014   0.185
E Q  5E6PA                417    0.513  91.604   1.361   0.923   0.002   0.076
B F  5E6PA                418    0.153  30.647   0.554   0.923   0.002   0.076
B V  5E6PA                419    0.049   7.547   0.643   0.970   0.001   0.030
E D  5E6PA                420    0.385  55.406   0.530   0.970   0.001   0.030
E N  5E6PA                421    0.463  67.725   0.484   0.988   0.000   0.012
B F  5E6PA                422    0.059  11.902   0.768   0.988   0.000   0.012
B F  5E6PA                423    0.053  10.657   0.939   0.970   0.001   0.030
E Q  5E6PA                424    0.376  67.064   1.550   0.970   0.001   0.030
B S  5E6PA                425    0.133  15.564   0.300   0.938   0.007   0.055
B V  5E6PA                426    0.059   9.068   0.295   0.938   0.007   0.055
B L  5E6PA                427    0.104  19.097   0.019   0.717   0.014   0.269
E A  5E6PA                428    0.401  44.234  -0.247   0.339   0.016   0.645
E P  5E6PA                429    0.347  49.253   0.082   0.109   0.005   0.886
E G  5E6PA                430    0.527  41.483  -1.609   0.016   0.005   0.979
E H  5E6PA                431    0.503  91.459  -1.124   0.016   0.005   0.979
E A  5E6PA                432    0.505  55.695  -0.496   0.053   0.005   0.942
B V  5E6PA                433    0.113  17.353   0.426   0.176   0.004   0.820
B P  5E6PA                434    0.124  17.624  -0.162   0.321   0.003   0.675
B P  5E6PA                435    0.215  30.537   0.184   0.717   0.014   0.269
B A  5E6PA                436    0.066   7.317  -0.502   0.802   0.014   0.185
B V  5E6PA                437    0.045   6.963   0.800   0.938   0.007   0.055
E K  5E6PA                438    0.275  56.609   0.949   0.970   0.001   0.030
B Y  5E6PA                439    0.191  40.902   0.738   0.970   0.001   0.030
B F  5E6PA                440    0.037   7.386   0.537   0.970   0.001   0.030
B F  5E6PA                441    0.061  12.203   0.675   0.923   0.002   0.076
E D  5E6PA                442    0.274  39.527   0.037   0.858   0.002   0.139
B F  5E6PA                443    0.115  23.081   0.820   0.858   0.002   0.139
B L  5E6PA                444    0.054   9.851   0.645   0.858   0.002   0.139
B D  5E6PA                445    0.228  32.812   0.569   0.923   0.002   0.076
E E  5E6PA                446    0.503  87.874   0.953   0.923   0.002   0.076
B Q  5E6PA                447    0.126  22.432   0.350   0.923   0.002   0.076
B A  5E6PA                448    0.034   3.791  -1.900   0.923   0.002   0.076
E E  5E6PA                449    0.599 104.663   0.367   0.858   0.002   0.139
E K  5E6PA                450    0.674 138.601   0.905   0.694   0.003   0.303
E H  5E6PA                451    0.352  64.011   0.113   0.246   0.004   0.750
E D  5E6PA                452    0.748 107.729  -0.065   0.016   0.005   0.979
B I  5E6PA                453    0.308  56.888  -1.186   0.003   0.003   0.994
E R  5E6PA                454    0.552 126.477  -0.048   0.016   0.005   0.979
E D  5E6PA                455    0.404  58.274   0.704   0.053   0.005   0.942
E E  5E6PA                456    0.676 118.132   0.604   0.502   0.002   0.495
E D  5E6PA                457    0.516  74.370  -0.402   0.522   0.016   0.462
B T  5E6PA                458    0.076  10.583   1.277   0.649   0.163   0.188
B I  5E6PA                459    0.127  23.514   1.056   0.578   0.229   0.194
E H  5E6PA                460    0.381  69.213   0.083   0.662   0.225   0.113
B I  5E6PA                461    0.154  28.472   0.675   0.662   0.225   0.113
B W  5E6PA                462    0.089  21.308   0.419   0.538   0.173   0.289
E K  5E6PA                463    0.388  79.750   0.222   0.278   0.093   0.628
E T  5E6PA                464    0.366  50.723  -1.173   0.191   0.086   0.723
B N  5E6PA                465    0.287  41.988  -1.395   0.053   0.043   0.903
B S  5E6PA                466    0.141  16.490  -1.362   0.053   0.043   0.903
B L  5E6PA                467    0.098  17.999   0.191   0.191   0.086   0.723
B P  5E6PA                468    0.107  15.240  -0.321   0.278   0.093   0.628
B L  5E6PA                469    0.065  11.828  -0.406   0.428   0.171   0.402
B R  5E6PA                470    0.189  43.258  -0.087   0.538   0.173   0.289
B F  5E6PA                471    0.077  15.494  -0.205   0.578   0.229   0.194
B W  5E6PA                472    0.040   9.548   0.023   0.594   0.285   0.121
B V  5E6PA                473    0.061   9.330   0.029   0.594   0.285   0.121
B N  5E6PA                474    0.147  21.594  -0.176   0.578   0.229   0.194
B I  5E6PA                475    0.063  11.692   0.017   0.428   0.171   0.402
B L  5E6PA                476    0.067  12.286  -0.560   0.307   0.165   0.527
E K  5E6PA                477    0.402  82.691   0.053   0.113   0.043   0.844
E N  5E6PA                478    0.291  42.632  -0.415   0.018   0.019   0.964
B P  5E6PA                479    0.121  17.127   0.017   0.018   0.019   0.964
E H  5E6PA                480    0.442  80.436  -0.107   0.019   0.141   0.840
B F  5E6PA                481    0.103  20.592  -0.327   0.022   0.552   0.426
B I  5E6PA                482    0.098  18.093  -0.109   0.018   0.846   0.136
B F  5E6PA                483    0.074  14.932  -0.010   0.018   0.846   0.136
B D  5E6PA                484    0.216  31.054  -0.302   0.021   0.756   0.223
B V  5E6PA                485    0.055   8.500   0.532   0.022   0.552   0.426
E H  5E6PA                486    0.460  83.765   0.673   0.021   0.451   0.528
E V  5E6PA                487    0.415  63.785   0.241   0.020   0.205   0.775
E H  5E6PA                488    0.308  55.971  -0.504   0.053   0.043   0.903
E E  5E6PA                489    0.577 100.854  -0.449   0.113   0.043   0.844
E V  5E6PA                490    0.279  42.852   0.123   0.191   0.086   0.723
B V  5E6PA                491    0.086  13.234   0.041   0.455   0.046   0.498
E D  5E6PA                492    0.319  45.997  -0.723   0.622   0.015   0.363
B A  5E6PA                493    0.187  20.596   0.098   0.802   0.014   0.185
B S  5E6PA                494    0.037   4.325  -0.729   0.938   0.007   0.055
B L  5E6PA                495    0.050   9.228   0.824   0.975   0.003   0.022
B S  5E6PA                496    0.224  26.300   0.071   0.975   0.003   0.022
B V  5E6PA                497    0.071  10.913   0.410   0.988   0.000   0.012
B I  5E6PA                498    0.025   4.680   0.679   0.988   0.000   0.012
B A  5E6PA                499    0.052   5.785  -0.202   0.970   0.001   0.030
B Q  5E6PA                500    0.246  43.953   0.191   0.970   0.001   0.030
B T  5E6PA                501    0.049   6.796   0.493   0.923   0.002   0.076
B F  5E6PA                502    0.037   7.366  -0.370   0.923   0.002   0.076
B M  5E6PA                503    0.184  36.738  -0.121   0.858   0.002   0.139
E D  5E6PA                504    0.307  44.253  -0.770   0.694   0.003   0.303
B A  5E6PA                505    0.056   6.215  -1.114   0.622   0.015   0.363
B C  5E6PA                506    0.062   8.719  -0.869   0.522   0.016   0.462
E T  5E6PA                507    0.428  59.308   0.051   0.354   0.048   0.598
E R  5E6PA                508    0.370  84.661  -0.664   0.278   0.093   0.628
B T  5E6PA                509    0.244  33.773  -0.357   0.191   0.086   0.723
E E  5E6PA                510    0.392  68.552   0.136   0.199   0.152   0.649
E H  5E6PA                511    0.321  58.317  -0.522   0.199   0.152   0.649
E K  5E6PA                512    0.535 110.152   0.450   0.199   0.152   0.649
B L  5E6PA                513    0.228  41.783  -0.157   0.191   0.086   0.723
E S  5E6PA                514    0.454  53.185  -1.206   0.113   0.087   0.800
E R  5E6PA                515    0.470 107.630  -0.984   0.053   0.043   0.903
E D  5E6PA                516    0.569  82.007  -1.940   0.053   0.043   0.903
B S  5E6PA                517    0.218  25.573  -0.939   0.018   0.019   0.964
E P  5E6PA                518    0.407  57.739  -0.989   0.053   0.043   0.903
E S  5E6PA                519    0.344  40.293  -1.463   0.053   0.043   0.903
E N  5E6PA                520    0.372  54.388  -1.370   0.113   0.043   0.844
E K  5E6PA                521    0.369  75.821   0.458   0.184   0.043   0.773
B L  5E6PA                522    0.179  32.702   0.321   0.191   0.086   0.723
B L  5E6PA                523    0.153  27.923   0.367   0.191   0.086   0.723
B Y  5E6PA                524    0.162  34.705  -0.146   0.278   0.093   0.628
B A  5E6PA                525    0.231  25.489  -0.108   0.278   0.093   0.628
E K  5E6PA                526    0.542 111.510   0.146   0.268   0.043   0.689
E E  5E6PA                527    0.457  79.855  -0.449   0.257   0.016   0.727
B I  5E6PA                528    0.130  24.069   1.292   0.339   0.016   0.645
E S  5E6PA                529    0.447  52.447  -0.327   0.502   0.002   0.495
E T  5E6PA                530    0.482  66.895   0.684   0.600   0.003   0.397
B Y  5E6PA                531    0.088  18.870   0.210   0.782   0.003   0.216
B K  5E6PA                532    0.237  48.771   0.818   0.923   0.002   0.076
E K  5E6PA                533    0.644 132.368   1.072   0.923   0.002   0.076
B M  5E6PA                534    0.237  47.444   0.946   0.923   0.002   0.076
B V  5E6PA                535    0.023   3.550   0.727   0.970   0.001   0.030
E E  5E6PA                536    0.467  81.602   0.915   0.970   0.001   0.030
E D  5E6PA                537    0.537  77.396   1.516   0.970   0.001   0.030
B Y  5E6PA                538    0.086  18.378   0.949   0.970   0.001   0.030
B Y  5E6PA                539    0.083  17.758   0.278   0.970   0.001   0.030
E K  5E6PA                540    0.565 116.220   1.845   0.970   0.001   0.030
E G  5E6PA                541    0.445  34.990   1.137   0.923   0.002   0.076
B I  5E6PA                542    0.042   7.751   0.227   0.858   0.002   0.139
E R  5E6PA                543    0.501 114.729   0.842   0.694   0.003   0.303
E Q  5E6PA                544    0.772 137.951   0.791   0.321   0.003   0.675
E M  5E6PA                545    0.298  59.630   0.429   0.176   0.004   0.820
B V  5E6PA                546    0.236  36.273  -0.562   0.053   0.005   0.942
E Q  5E6PA                547    0.585 104.427   0.493   0.018   0.019   0.964
B V  5E6PA                548    0.152  23.409  -0.881   0.005   0.015   0.979
E S  5E6PA                549    0.411  48.204   0.289   0.005   0.015   0.979
E D  5E6PA                550    0.518  74.673  -0.368   0.782   0.003   0.216
E Q  5E6PA                551    0.691 123.484   0.791   0.923   0.002   0.076
E D  5E6PA                552    0.412  59.441   1.142   0.923   0.002   0.076
B M  5E6PA                553    0.054  10.825   0.741   0.970   0.001   0.030
E N  5E6PA                554    0.377  55.120   1.126   0.970   0.001   0.030
E T  5E6PA                555    0.610  84.579   1.378   0.970   0.001   0.030
B H  5E6PA                556    0.241  43.838   0.575   0.970   0.001   0.030
B L  5E6PA                557    0.039   7.068   0.420   0.970   0.001   0.030
E A  5E6PA                558    0.354  39.022   1.257   0.970   0.001   0.030
E E  5E6PA                559    0.611 106.689   1.311   0.970   0.001   0.030
B I  5E6PA                560    0.130  24.124   0.611   0.970   0.001   0.030
B S  5E6PA                561    0.054   6.376  -0.610   0.923   0.002   0.076
E R  5E6PA                562    0.544 124.507   0.417   0.923   0.002   0.076
E A  5E6PA                563    0.601  66.263   1.120   0.923   0.002   0.076
B H  5E6PA                564    0.135  24.556   0.130   0.858   0.002   0.139
E T  5E6PA                565    0.377  52.290  -0.489   0.694   0.003   0.303
E D  5E6PA                566    0.566  81.546  -1.593   0.502   0.002   0.495
B S  5E6PA                567    0.298  34.972   0.073   0.600   0.003   0.397
B L  5E6PA                568    0.157  28.765  -0.278   0.694   0.003   0.303
E N  5E6PA                569    0.342  50.069  -0.480   0.694   0.003   0.303
B T  5E6PA                570    0.205  28.489   0.086   0.923   0.002   0.076
B L  5E6PA                571    0.133  24.297  -0.324   0.923   0.002   0.076
B V  5E6PA                572    0.123  18.967  -1.341   0.970   0.001   0.030
B A  5E6PA                573    0.054   5.984  -0.401   0.970   0.001   0.030
B L  5E6PA                574    0.034   6.244  -0.050   0.970   0.001   0.030
E H  5E6PA                575    0.283  51.532   0.534   0.988   0.000   0.012
E Q  5E6PA                576    0.258  46.132   0.665   0.988   0.000   0.012
B L  5E6PA                577    0.026   4.742   0.676   0.988   0.000   0.012
B Y  5E6PA                578    0.114  24.362   0.621   0.970   0.001   0.030
E Q  5E6PA                579    0.489  87.425   1.159   0.970   0.001   0.030
B Y  5E6PA                580    0.191  40.902   0.737   0.923   0.002   0.076
B T  5E6PA                581    0.036   4.979  -0.281   0.879   0.010   0.111
E Q  5E6PA                582    0.455  81.192   0.971   0.717   0.014   0.269
E K  5E6PA                583    0.551 113.402   1.161   0.600   0.003   0.397
B Y  5E6PA                584    0.273  58.383   0.191   0.406   0.004   0.590
B Y  5E6PA                585    0.198  42.334   0.550   0.782   0.003   0.216
E D  5E6PA                586    0.601  86.561  -0.174   0.858   0.002   0.139
E E  5E6PA                587    0.465  81.183   1.156   0.938   0.007   0.055
B I  5E6PA                588    0.025   4.607   0.865   0.938   0.007   0.055
B I  5E6PA                589    0.188  34.706   0.649   0.923   0.002   0.076
E N  5E6PA                590    0.594  86.903   1.155   0.923   0.002   0.076
B A  5E6PA                591    0.161  17.720   0.145   0.858   0.002   0.139
B L  5E6PA                592    0.125  22.979   0.507   0.802   0.014   0.185
E E  5E6PA                593    0.601 104.942   0.068   0.522   0.016   0.462
E E  5E6PA                594    0.595 103.912   0.020   0.339   0.016   0.645
E D  5E6PA                595    0.327  47.063   0.300   0.053   0.005   0.942
E P  5E6PA                596    0.649  92.065   0.089   0.694   0.003   0.303
E A  5E6PA                597    0.548  60.379   0.021   0.858   0.002   0.139
B A  5E6PA                598    0.089   9.775  -1.305   0.858   0.002   0.139
E Q  5E6PA                599    0.308  54.991  -0.028   0.923   0.002   0.076
E K  5E6PA                600    0.521 107.129   0.188   0.858   0.002   0.139
E M  5E6PA                601    0.383  76.738   0.264   0.782   0.003   0.216
E Q  5E6PA                602    0.462  82.495   0.177   0.782   0.003   0.216
B L  5E6PA                603    0.092  16.790   0.310   0.858   0.002   0.139
E A  5E6PA                604    0.322  35.506  -0.157   0.923   0.002   0.076
E F  5E6PA                605    0.405  81.223  -0.588   0.970   0.001   0.030
E R  5E6PA                606    0.278  63.548   1.162   0.970   0.001   0.030
B L  5E6PA                607    0.055   9.997   0.665   0.988   0.000   0.012
E Q  5E6PA                608    0.393  70.136   0.971   0.988   0.000   0.012
E Q  5E6PA                609    0.338  60.278   1.353   0.988   0.000   0.012
B I  5E6PA                610    0.032   5.883   0.464   0.988   0.000   0.012
B A  5E6PA                611    0.101  11.163   0.161   0.988   0.000   0.012
E A  5E6PA                612    0.377  41.512   0.347   0.970   0.001   0.030
B A  5E6PA                613    0.137  15.130   0.577   0.923   0.002   0.076
B L  5E6PA                614    0.089  16.296   0.434   0.858   0.002   0.139
E E  5E6PA                615    0.475  82.895   0.428   0.694   0.003   0.303
E N  5E6PA                616    0.553  80.959  -0.759   0.406   0.004   0.590
E K  5E6PA                617    0.559 114.986  -0.062   0.321   0.003   0.675
B V  5E6PA                618    0.267  41.069  -1.215   0.257   0.016   0.727
E T  5E6PA                619    0.492  68.240  -0.507   0.181   0.016   0.803
E D  5E6PA                620    0.644  92.858  -0.852   0.058   0.017   0.925
E L  5E6PA                621    0.540  98.929  -1.186   0.003   0.003   0.994

Explain the output.  Go back.
```

---
